# Emergence of difficult-to-treat lymphocutaneous sporotrichosis? Analysis based on three cases with high and low MIC *Sporothrix brasiliensis* isolates

**DOI:** 10.1590/S1678-9946202668041

**Published:** 2026-07-03

**Authors:** Juliana Carreiro Avila, Ana Paula Carvalho Reis, Kaique Arriel, Amanda Azevedo Bittencourt, Livia Vieira Almeida, Rita de Cassia Soler, Giovanna Azevedo Celestrino, Ana Paula Cordeiro Lima, Maria Gloria Teixeira Sousa, José Angelo Lauletta Lindoso, Gil Benard

**Affiliations:** 1 Universidade de São Paulo, Faculdade de Medicina, Instituto de Medicina Tropical de São Paulo, Laboratório de Micologia Médica (LIM-53), São Paulo São Paulo, Brazil; 2 Instituto de Infectologia Emilio Ribas, São Paulo, São Paulo, Brazil

**Keywords:** Sporotrichosis, Sporothrix brasiliensis, Itraconazole, Terbinafine

## Abstract

Sporotrichosis, caused by a *Sporothrix schenckii* complex species, is historically viewed as an easily manageable mycosis. However, the current zoonotic epidemic in Brazil due to the more virulent *Sporothrix brasiliensis* poses some challenges. We describe three cases of lymphocutaneous sporotrichosis (LC) in non-immunocompromised patients, which proved refractory to standard treatment, and suggest that closer epidemic surveillance would be advisable to detect whether more difficult-to-treat cases are ­­emerging. All cases required prolonged treatment, escalating doses, and therapeutic changes for resolution. Patients’ isolates were molecularly identified as *S. brasiliensis*. Antifungal susceptibility testing (AST) revealed high itraconazole MICs in two of the three isolates. Management required high-dose itraconazole (400 mg/day) in two cases and Amphotericin B in one. These cases challenge the perception of sporotrichosis as an always easily treatable disease. The difficulties observed may be linked to the increased *S. brasiliensis* virulence, the emergence of drug-resistant strains, and/or the rise in severe and atypical presentations. Lack of standardized protocols for refractory cases complicates effective management. These findings underscore the urgent need for enhanced epidemiological surveillance, efforts to isolate and perform AST on clinical strains, and standardization of treatment guidelines for patients failing initial therapy.

## INTRODUCTION

Sporotrichosis was first described in 1898 by Benjamin Schenck, at the time a medical student at John Hopkins. In Brazil, the first case was reported by Lutz and Splendore in 1907. This subcutaneous mycosis is historically associated with traumatic cutaneous inoculation of dimorphic fungi of the *Sporothrix* genus which are found in soil, plants, and decaying organic matter. However, zoonotic infections transmitted via bites, scratches, and contact with lesion exudates of infected animals, especially cats, have dramatically increased. This caused an epidemiological shift of the disease over the last twenty years, especially in Brazil, from a work-associated mycosis of farmers and gardeners to a predominantly zoonotic disease, becoming an increasing public health concern^1^.

Until the early 2000's, sporotrichosis was thought to be caused by a single species, *Sporothrix schenckii*, but new species have been identified, including *Sporothrix schenckii* sensu strictu, *S. globosa, S. mexicana*, *S. luriei*, and *S. brasiliensis*, making up the *Sporothrix schenkii* complex^2^. This latter species, proposed in 2007, was responsible for the feline and human sporotrichosis epidemic outbreaks that started in Rio de Janeiro, in the 1990's, but now has spread across the country and to other South American countries like Paraguay, Argentina, Chile^3^. The Brazilian epidemic is characterized by: a) an up to now uncontrolled zoonotic transmission, predominantly cat-to-cat and cat-to-human, mainly through bites and scratches^4^; and b) a new, morphologically undistinguishable species that showed experimentally higher virulence^5^. However, a recent retrospective study revealed that some epidemic cases could be due to non-zoonotic transmission, with the route of infection being mainly trauma involving plants and/or contact with soil^6^. Virulence mechanisms might be associated with differences in cell wall structure^7^, cell morphometry, cell wall topography, and gp70 expression^8^.

Historically, sporotrichosis has been considered a disease mostly localized on the skin or on the skin and subcutaneous tissue, and generally not representing a challenge to treat^9^. In fact, open treatment trials in which itraconazole was given at dosages of 100 to 200 mg a day for two to nine months have reported success rates of 90% to 100%, without significant adverse events^10-12^. Clinical improvement usually occurred in the first four weeks of treatment, and only a very small percentage of patients needed higher itraconazole dosages or other medication^9^.

Here, we describe three cases of lymphocutaneous (LC) sporotrichosis in non-immunosuppressed patients which posed a challenge for conventional treatment. These cases underpins our hypothesis that the historically favorable treatment scenario for human sporotrichosis should be reevaluated in the coming years.

## MATERIAL AND METHODS

### Ethics

This study was approved by the Ethics Committee of the Emilio Ribas Infectology Institute (IIER), CAAE Nº 65476822.6.3001.0061. All donors provided written informed consent.

### Isolates identification and antifungal susceptibility testing

Samples were obtained from patients’ skin lesions and inoculated onto Sabouraud dextrose agar for 7–10 days. Fungal growth was evaluated by direct microscopy to identify typical *Sporothrix* spp. microconidia. All isolates were characterized down to species level using a species-specific PCR assay previously described by Rodrigues *et al*.^13^, which targets fragments of the calmodulin gene. Antifungal susceptibility testing for the mycelial form of *Sporothrix* spp was performed by broth dilution according to EUCAST document E.DEF 9.4. Plates were incubated at 30 °C, the quality controls were read at 48 h, and the clinical isolates at 72 h. The antifungal drugs used consisted of itraconazole, terbinafine, fluconazole, and amphotericin B (all purchased from Sigma-Aldrich, Burlington, MA, USA). Minimum inhibitory concentrations (MICs) were determined by visual inspection of complete growth inhibition, and of fungal growth compared with the controls. All assay runs included *Aspergillus flavus* ATCC 204304 and *Aspergillus fumigatus* ATCC 204305 as quality controls^14^,^15^.

### Patients description

**Figure 1:**
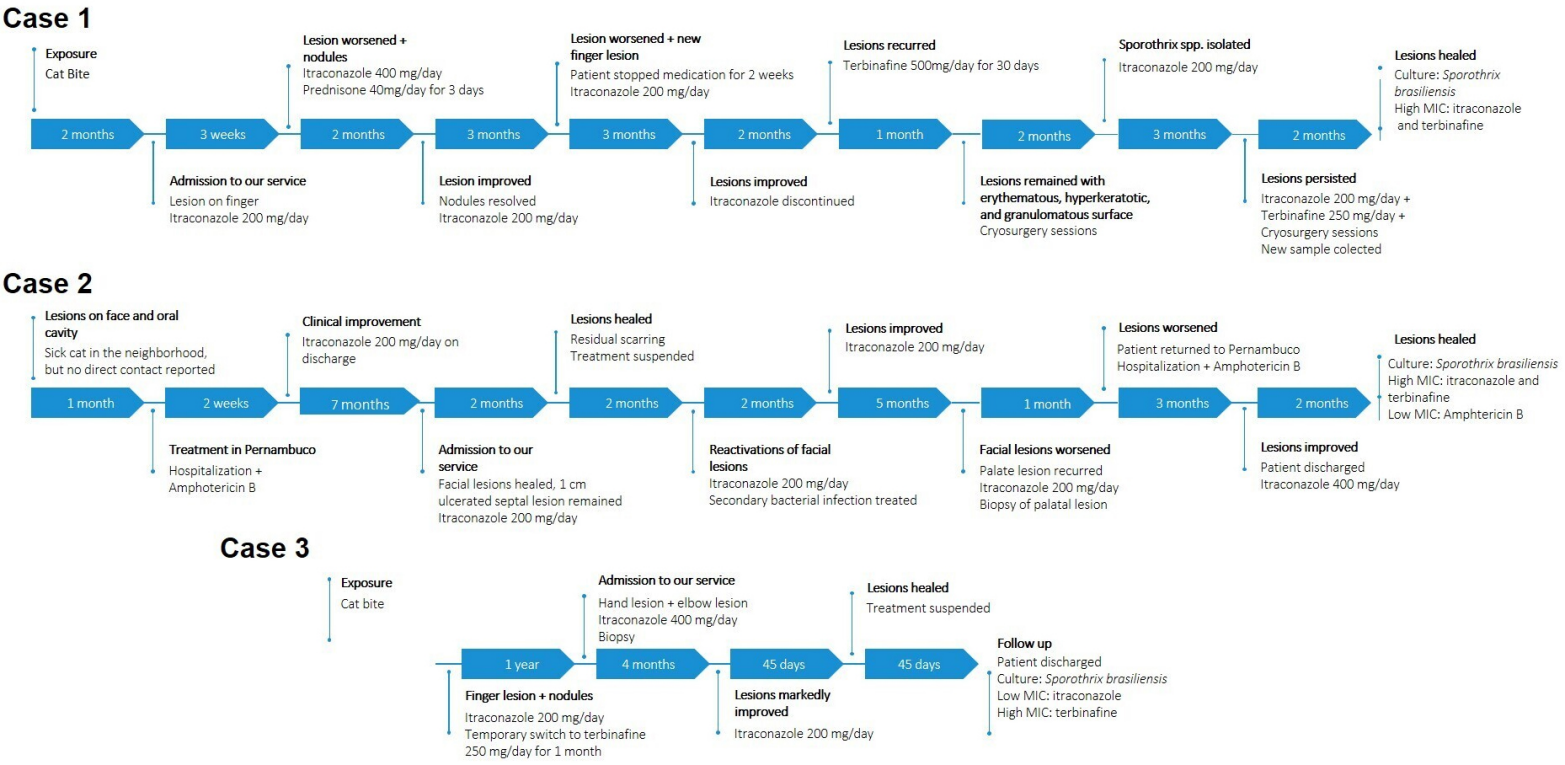
Timelines with the main events of the patients’ clinical evolution.

Figure 1 illustrates the timelines with the main events in the clinical evolution of the three patients.

#### CASE 1

**Figure 2:**
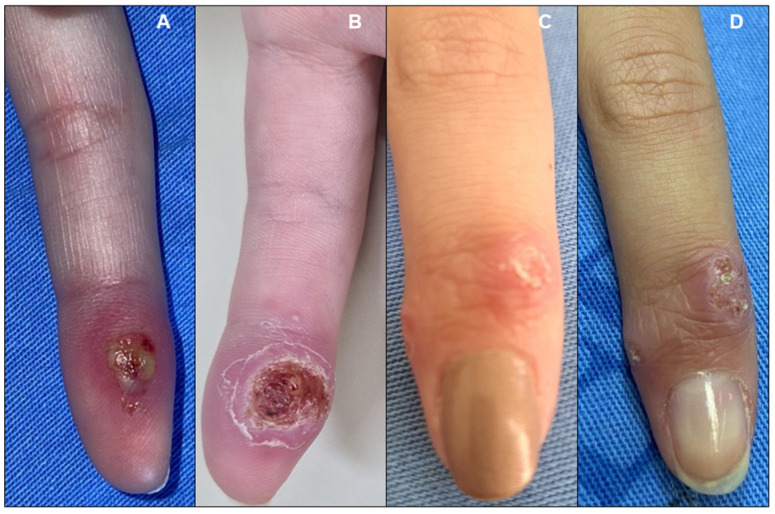
Case 1: (A) Pulp finger lesion on the first visit, two months after having been bitten by a cat; (B) Worsening of pulp finger lesion after 3 weeks of itraconazole 200 mg/day; (C) New lesion on the dorsum of the right index finger after 3 months of follow-up on itraconazole, but with itraconazole interruption by the patient in the prior 2 weeks; (D) Worsening of dorsal finger lesion after suspension of the second course of itraconazole.

A 24-year-old female from Sao Paulo city, Brazil, presented to us with a small abscess on the digital pulp of her right second finger (Figure 2A), where she had been bitten two months before by a cat with sporotrichosis. No other lesions were noted. After a diagnosis of fixed cutaneous sporotrichosis, itraconazole 200 mg/day was prescribed to the patient. Three weeks later, the lesion worsened, becoming ulcerated (Figure 2B), and subcutaneous nodules appeared on the right forearm, accompanied by fever. The diagnosis was changed to lymphocutaneous sporotrichosis, and the itraconazole dosage was increased to 400 mg/day. Prednisone 40 mg/day was also prescribed for three days. After two months, the patient's right finger lesion had improved, and the nodules in the right forearm had resolved. Itraconazole was then reduced to 200 mg/day.

Three months later, however, the patient returned with a new lesion on the dorsum of the right index finger (Figure 2C). She had stopped itraconazole for the previous two weeks. Itraconazole 200 mg/day was reintroduced. After three months, this new lesion was much improved, presenting only mild superficial scaling. Itraconazole was then suspended. At the next visit, 2 months later, terbinafine was prescribed (500 mg/day for 30 days) due to the recrudescence of this dorsal lesion (Figure 2D). With this treatment, both the first pulp lesion and the second dorsal lesion developed a hyperkeratotic and erythematous aspect with a granulomatous surface. The patient was then treated with cryosurgery sessions alone for two months, after which a scraping of the lesions was performed, yielding *Sporothrix spp*. With this finding, itraconazole 200 mg/day was reintroduced. Because the patient returned three months after itraconazole reintroduction still with active lesions, a new sample was collected for culture, and terbinafine (250 mg/day) was added to the treatment, together with additional cryosurgery sessions. After several sessions, the lesions finally healed. The sample culture still resulted in *Sporothrix spp*. growth. Molecular analysis confirmed the species as *brasiliensis*. AST showed that the isolate had high MICs to both itraconazole and terbinafine (Table 1).

**Table 1:** Susceptibility profile of *Sporothrix brasiliensis* isolates

Antifungal	MIC (mg/L)		
	Case 1	Case 2	Case 3
Amphotericin B	2	2	1
Itraconazole	> 8	> 8	0.25
Terbinafine	4	2	1
Fluconazole	> 64	> 64	> 64

#### CASE 2

**Figure 3:**
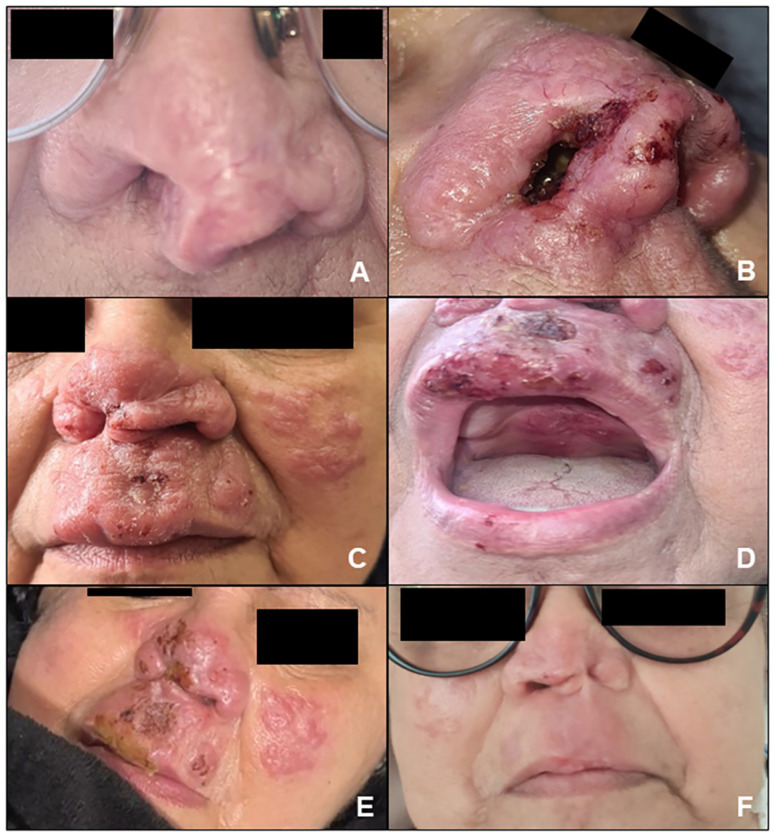
Case 2: (A) Nasal lesion with a healed appearance on admission (there was however a small [∼1cm] ulcerated septal lesion with a granulomatous surface); (B) Worsening of lesions with edema and erythema of the nose, crusts, and a purulent secretion and signs of secondary infection after two months of follow-up with itraconazole 200 mg/day; (C) New recrudescence of the granulomatous nasal lesions, this time accompanied by relapse of the granulomatous lesion on the palate; (D) After 7 months of follow-up; (E) Still active lesions by the time the patient left our service; (F) Resolution of lesions after treatment with amphotericin B followed by itraconazole 400 md/day in another service.

The patient, a 65-year-old female residing in a rural area in Pernambuco State, Brazil, presented to our clinic due to a nine-month history of facial skin lesions (lips, peri-labial, and nose), but also involving the oral cavity. She had previously sought medical care at her home state. At that time, the patient's biopsies of palate and lip lesions showed a chronic granulomatous inflammatory process, while Grocott staining exhibited multiple intracellular spherical fungal-like structures within macrophages. Sporotrichosis is highly endemic in Pernambuco. Additionally, there was a sick cat in the neighborhood, but the patient denied close contact with it. Lymphocutaneous sporotrichosis was considered and, due to the severity of the lesions, the patient was hospitalized to receive treatment with deoxycholate amphotericin B. The lesions improved and she was discharged to receive itraconazole 200 mg/day, which she had been taking for the past seven months, showing further improvement. She then moved to Sao Paulo city for treatment follow-up. On admission, the facial lesions appeared healed (Figure 3A), except for a small (∼1 cm) ulcerated septal lesion with a granulomatous surface. Due to the apparent good response, itraconazole 200 mg/day was maintained for another two months, after which she returned with healed lesions, persisting only the scarring aspect, and the therapy was suspended.

Two months later, however, the patient returned with partially reactivated lesions: edema and erythema of the nose, crusts, and a purulent secretion (Figure 3B). The secondary bacterial infection was treated with cotrimoxazole and resolved. Itraconazole was prescribed again, with an initial good response after two months.

However, after seven months of treatment, she again experienced worsening of the granulomatous nasal lesions (Figure 3C) and relapse of the granulomatous lesion on the palate (Figure 3D). Biopsy of the latter evinced granulomatous inflammation with very rare intracellular yeast cells identified by PAS, but not by Grocott staining (probably due to the ongoing antifungal treatment). Other staining methods (Ziehl-Neelsen and Giemsa) were negative. Serological testing for histoplasmosis and histochemistry tests for *Leishmania* spp., *Histoplasma* spp., and *Paracoccidioides* spp. antigens were also negative. Culture yielded *Sporothrix* spp., which was molecularly identified as *S. brasiliensis*. Antifungal susceptibility testing showed that the fungus presented high MICs for both itraconazole and terbinafine, but low MIC for Amphotericin B (Table 1). A subsequent *in situ* PCR was also positive for *S. brasiliensis*. However, at this time, the patient returned to her home state.

There, with progressing lesions despite 200 mg/day itraconazole treatment (Figure 3E), she was hospitalized to receive amphotericin B deoxycholate for three months (she required several treatment interruptions due to adverse renal effects), followed by itraconazole 400 mg/day for another two months, with complete resolution of the facial lesions (Figure 3F). Since then (around eighteen months) no relapses of these lesions were reported by the patient.

#### CASE 3

A 62-year-old female patient, residing in Sao Paulo city's metropolitan area, reported a one-year history of skin lesions on her right arm. The lesions, which appeared one month after the patient had been bitten at the same site by a stray cat with cutaneous lesions, began as a pustule on the right hand that progressed to an ulcerated lesion. The patient also noted the appearance of subcutaneous nodules (up to 2 cm) in line with and adjacent to the cutaneous lesion, some of which became ulcerated but soon healed. She went to a dermatologist consultant who, based on the clinical presentation and epidemiological link, diagnosed LC sporotrichosis and started treatment with itraconazole 200 mg/day. After a total of four consultations and one year of regular intake of the medication (including one month of terbinafine 250 mg/day replacing itraconazole) without improvement (rather, the lesion slowly but gradually enlarged and became painful), she came to our service.

**Figure 4:**
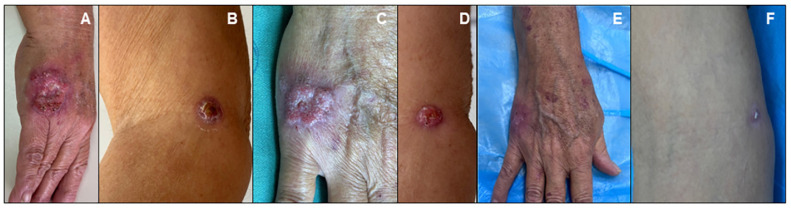
Case 3: (A) A 5 cm diameter ulcerated verrucous plaque on the dorsum of the right hand; (B) A small plaque (1.2 cm) on the medial side of the right elbow, on admission, despite 1 year of itraconazole 200 mg/day; (C and D) Lesions with significant improvement after 1 month of itraconazole 400 mg/day; (E and F) Further lesion improvement after 4 months of itraconazole 400 mg/day.

On admission, physical examination noted a 5 cm ulcerated verrucous plaque on the dorsum of the right hand (Figure 4A) and a smaller 1.2 cm plaque on the medial side of the right elbow (Figure 4B). No nodules were palpable at the time. A biopsy was scheduled and, meanwhile, itraconazole dosage was raised to 400 mg/day. The biopsy revealed a dermal mixed inflammatory process with rare yeasts of varying sizes and budding. Fungal culture yielded *Sporothrix spp.*, which was molecularly identified as *S. brasiliensis*. Susceptibility testing showed that the isolate presented a low MIC for itraconazole and a high MIC for terbinafine (Table 1). During follow-up, both lesions showed significant improvement (Figures 4C and 4D). Itraconazole 400 mg/day was therefore maintained. After four months of itraconazole 400 mg/day, due to the highly favorable clinical response (Figures 4E and 4F), the dosage was reduced to 200 mg/day for another 45 days and then suspended. No relapse has been reported by the patient up to now.

## DISCUSSION

We presented three LC sporotrichosis cases that challenged conventional treatment, requiring multiple retreatments with varying antifungal doses and/or changes in antifungal drugs. Two cases were eventually related to the high MICs in patients’ isolates; however, the third was caused by a "wild type" isolate (Table 1). None of the patients had a past history suggestive of immunocompromise or comorbidities, and all tested negative on HIV serology.

High response rates with itraconazole treatment were initially reported in the Brazilian epidemics. A study with 645 patients treated with this antifungal in Rio de Janeiro state between 2002 and 2006 reported an overall cure rate of 94.6% after a median treatment duration of twelve weeks^16^. Remarkably, 90.3% were cured with a 100 mg dose, and the remaining with 200–400 mg. Of the latter, only 1.4% required retreatment with another antifungal drug or thermotherapy due to relapse or adverse effects, and all patients were successfully retreated. The other 4.0% abandoned treatment.

Guidelines and technical notes on sporotrichosis treatment have been published in the last 20 years. The 2007 Infectious Diseases Society of America ("IDSA") guideline recommends the FC/LC forms itraconazole 200 mg/day as first-line treatment for three to six months^9^. For non-responsive patients, options include itraconazole 400 mg/day, terbinafine 1 g/day, or potassium iodide. However, it does not address the Brazilian epidemic. The European Confederation of Medical Mycology maintained the same general recommendations^17^. More recently, the Brazilian Society of Dermatology released a guideline also proposing itraconazole as first-line treatment, but at daily doses of 100–200 mg, to treat the FC/LC forms^18^. Alternatives include terbinafine (at lower doses than recommended by the IDSA: 250–500 mg/day), potassium iodide and, less frequently, amphotericin B^18^. Treatment duration was not clearly defined, ranging "from one to 12 months or longer, with a mean of 3 to 4 months"^18^. Differently from the ISDA guideline, the Brazilian Society recommends against the necessity of prolonging treatment for two to four weeks after lesion resolution^18^.

The Sao Paulo Health Municipality technical note^19^, in turn, recommends an itraconazole dosing schedule based on the clinical form: LC form should initiate with 400 mg/day followed by 200 mg/day, whereas the FC form should initiate with 200 mg/day followed by 100 mg/day. Conversely, other regional protocols generally recommend itraconazole 100–200 mg/day for three to six months or more^20-22^. A major point of contention is the post-resolution treatment duration: while some technical notes recommend continuation for three to four weeks after clinical cure, others do not. One technical note also mentions terbinafine (250–500 mg/day) as an alternative for patients who have a contraindication to itraconazole. In short, initial treatment doses and duration vary between different guidelines and technical notes.

Additionally, there are no clear recommendations for how to manage patients with cutaneous sporotrichosis who do not respond to the proposed itraconazole doses. The IDSA guideline mentions that itraconazole doses can be increased or the drug switched to terbinafine 1g/day or potassium iodide^9^. The Brazilian Society of Dermatology guideline proposes 250–500 g of terbinafine or potassium iodide^18^. Both documents fail to give a clear definition of what constitutes treatment failure, and a clear indication of how long treatment is needed before one should increase the itraconazole dose or change the antifungal class.

This scenario is further complicated by studies suggesting that the *S. brasiliensis* epidemic is associated with high rates of atypical forms of the mycosis. Such atypicality was first consistently observed in Rio de Janeiro; 50 patients with unusual presentations were identified from a cohort of 246 patients in whom the agent could be isolated and identified^23^. Unusual presentations consisted mainly of patients with disseminated cutaneous infections (but no underlying disease), hypersensitivity reactions, and mucosal infections. Several factors contributed to the higher incidence of such atypical cases amidst the *S. brasiliensis* epidemic compared with "classical" sporotrichosis due to *S. schenkii*. Among them were the size of the initial inoculum, the depth of the traumatic inoculation, and the eventual repeated inoculations due to daily exposure to cats (which can harbor high fungal burdens)—all features that distinctly characterize the current zoonotic epidemic from the sporadic *S. schenkii* infection^23^,^24^. Related to this are experimental observations of higher virulence of *S. brasiliensis* compared with other pathogenic species^25^. Importantly, in the aforementioned study on atypical presentations, initial response was unsatisfactory in 17.1% of the patients treated with itraconazole. These patients required more than twenty-four weeks of treatment and some additionally required increased doses (up to 400 mg). More recently, a systematic review of atypical cases, which included immunocompetent patients with ocular, nasal and oral mucosa involvement, and extracutaneous cases, highlighted the increasing burden posed by such patients. However, detailed data on their antifungal treatment was not provided^26^.

Another issue with the oral itraconazole treatment of sporotrichosis is its erratic absorption and bioavailability which might contribute to poor treatment responses, as clearly illustrated by the recent report of two patients with sporotrichosis who had previously undergone bariatric surgery and did not respond to itraconazole^27^. Unfortunately, as serum level monitoring was not available for our patients, this possibility could not be completely ruled out.

In addition to the rise of atypical cases, as perhaps our case 2 might be classified, another emerging concern regarding sporotrichosis treatment is the development of antifungal resistance. Early studies revealed that the strains associated with the Rio de Janeiro epidemic were highly susceptible to itraconazole, with similar MIC values being observed among isolates from different clinical forms of the mycosis; all isolates were collected from patients between 1999 and 2004^28^. Several studies were published since then with somewhat conflicting results. Some studies detected sizable proportions, reaching up to 37.5% of high itraconazole MICs among human and cats isolates from the epidemic, indicative of a rising trend for non-wild type isolates^29-31^. Conversely, others showed low rates of such high MIC isolates from humans^32^,^33^ and cats^34^. Notably, some of these studies collected isolates over a wide time period, mainly at the beginning of the epidemic, and may not reflect the cumulative exposure to itraconazole. However, some authors found no time-related increase in the rate of non-wild type isolates between 1999 and 2018^32^,^35^. The scenario regarding terbinafine is also variable, with both high^30^ and low MICs described^36^.

A limitation in these studies is that MIC breakpoints have not yet been definitively established for the *S. schenckii* complex. *Sporothrix* resistance has generally been inferred using epidemiological cutoff values (ECVs) based mostly on Clinical & Laboratory Standards Institute (CSLI) guidelines. In 2017, a multicenter study proposed *S. brasiliensis* ECVs of 2μg/mL for itraconazole and 0.12 μg/mL for terbinafine^14^. A Brazilian study in the same year also proposed this ECV for itraconazole, but suggested 0.25 μg/mL for terbinafine^37^.

A review on *S. brasiliensis* antifungal resistance pointed out that, despite the heterogeneous MIC results, emergence of *S. brasiliensis* with *in vitro* antifungal resistance seems likely. First, as illustrated by the reported cases, high antifungal exposure has taken place since the start of the epidemic. Second, *S. brasiliensis* shows high ability to develop resistance through several mechanisms such as melanin production, genetic diversity, and cytochrome P450 mutations^35^.

Unfortunately, clinical data on resistance is scarce. The few initial studies that attempted to correlate high MICs to refractory cases failed to demonstrate an association^32^. However, said association was shown in felines refractory to treatment, from which itraconazole-resistant *S. brasiliensis* were isolated^38^. A retrospective study provided indirect evidence by showing that 13% of patients’ isolates were non-wild type, most of whom required either longer than the standard three to six months of treatment or a higher itraconazole dose than the usually recommended 100–200 mg^39^. Only more recently were proven clinical and laboratory resistance concomitantly described in a LC patient^40^.

The cases described here further stress this concern: two were refractory to treatment, and their isolates showed high MICs for both itraconazole and terbinafine. Notably, although some studies suggest that resistance acquisition during prolonged antifungal treatment is unlikely in *S. brasiliensis* ^32^, resistance induction cannot be ruled out in our cases. Differently from our previous case, the isolates were obtained after the patients had initiated itraconazole treatment.

## CONCLUSION

The three cases described here—two with and one without high MICs—may pose a challenge to the historical perception that sporotrichosis is an easily treatable and non-complicated disease. More difficult-to-treat cases may emerge in the near future due to increased atypical and more severe cases, uncontrolled use of antifungals, development of resistant strains, and/or higher *S. brasiliensis* virulence. This underscores the need for closer epidemiological surveillance of the epidemic, as suggested by other reports^40^. Thus, growing efforts should be put in: (a) isolating the agent and performing AST for early detection of changes in the antifungal profile of *Sporothrix brasiliensis*; (b) reporting atypical cases or cases in which the proposed initial treatment failed to detect an eventual change in the clinical profile of the disease; (c) standardizing treatment protocols to allow valid comparison between them and provide better guidance for difficult-to-treat cases.

## Data Availability

The complete anonymized dataset supporting the findings of this study is included within the article itself.
